# Tri­chlorido­(1-ethyl­piperazin-1-ium)cobalt(II)

**DOI:** 10.1107/S1600536814006989

**Published:** 2014-04-05

**Authors:** Abdelhamid Chiheb Dhieb, Daron E. Janzen, Mohamed Rzaigui, Wajda Smirani Sta

**Affiliations:** a Laboratoire de Chimie des Matériaux, Faculté des Sciences de Bizerte, 7021 Zarzouna Bizerte, Tunisia; b Department of Chemistry and Biochemistry, St Catherine University, 2004 Randolph Avenue, #4282, St Paul, MN 55105, USA

## Abstract

In the title complex, [Co(C_6_H_15_N_2_)Cl_3_], the Co^2+^ ion is coordinated in a distorted tetra­hedral fashion by three chloride ions and one N atom of the piperazine ring; the ring adopts a chair conformation with the N—Co and N—C_Et_ bonds in equatorial orientations. In the crystal, mol­ecules are connected by N—H⋯Cl hydrogen bonds, generating (10-1) sheets.

## Related literature   

For related structures, see: Ciccarese *et al.* (1998 ▶); Clemente *et al.* (1999 ▶); Marzotto *et al.* (2000 ▶).
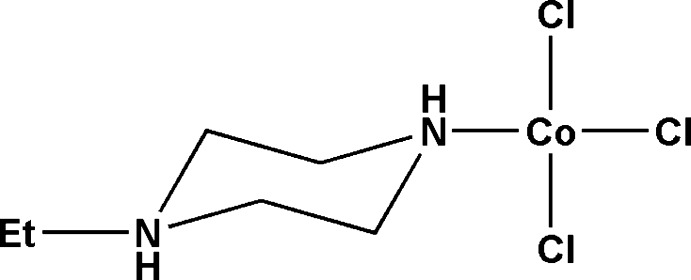


## Experimental   

### 

#### Crystal data  

[Co(C_6_H_15_N_2_)Cl_3_]
*M* *_r_* = 280.49
Monoclinic, 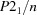

*a* = 7.421 (3) Å
*b* = 18.160 (7) Å
*c* = 8.691 (4) Å
β = 90.524 (7)°
*V* = 1171.1 (8) Å^3^
*Z* = 4
Mo *K*α radiation
μ = 2.10 mm^−1^
*T* = 173 K
0.32 × 0.13 × 0.13 mm

#### Data collection  

Rigaku XtaLAB mini diffractometer
Absorption correction: multi-scan (*REQAB*; Rigaku, 1998 ▶) *T*_min_ = 0.529, *T*_max_ = 0.761
11015 measured reflections
2399 independent reflections
2099 reflections with *F* ^2^ > 2σ(*F* ^2^)
*R*_int_ = 0.025

#### Refinement  

*R*[*F* ^2^ > 2σ(*F* ^2^)] = 0.022
*wR*(*F* ^2^) = 0.050
*S* = 1.08
2399 reflections
118 parameters
H atoms treated by a mixture of independent and constrained refinement
Δρ_max_ = 0.27 e Å^−3^
Δρ_min_ = −0.27 e Å^−3^

### 

Data collection: *CrystalClear-SM Expert* (Rigaku, 2011 ▶); cell refinement: *CrystalClear-SM Expert*; data reduction: *CrystalClear-SM Expert*; program(s) used to solve structure: *SIR2004* (Burla *et al.*, 2005 ▶); program(s) used to refine structure: *SHELXL97* (Sheldrick, 2008 ▶); molecular graphics: *ORTEP-3 for Windows* (Farrugia, 2012 ▶); software used to prepare material for publication: *CrystalStructure* (Rigaku, 2010 ▶).

## Supplementary Material

Crystal structure: contains datablock(s) General, I. DOI: 10.1107/S1600536814006989/hb7215sup1.cif

Structure factors: contains datablock(s) I. DOI: 10.1107/S1600536814006989/hb7215Isup2.hkl

CCDC reference: 994292

Additional supporting information:  crystallographic information; 3D view; checkCIF report

## Figures and Tables

**Table 1:** Selected bond lengths (Å)

Co1—Cl1	2.2720 (8)
Co1—Cl2	2.2419 (10)
Co1—Cl3	2.2691 (8)
Co1—N1	2.0686 (15)

**Table 2:** Hydrogen-bond geometry (Å, °)

*D*—H⋯*A*	*D*—H	H⋯*A*	*D*⋯*A*	*D*—H⋯*A*
N2—H2⋯Cl1^i^	0.854 (19)	2.347 (18)	3.1794 (16)	165.4 (15)
N1—H1⋯Cl3^ii^	0.83 (2)	2.49 (2)	3.3192 (17)	176.0 (17)

Symmetry codes: (i) 
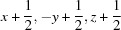
; (ii) 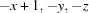.

**Fig. 1.**
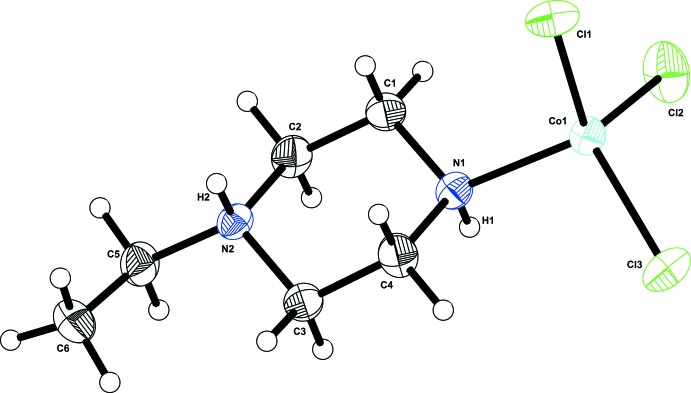
ORTEP-3 view of (C6H15N2)CoCl3 with displacement ellipsoids for non-H atoms drawn at the 30% probability level.

**Fig. 2.**
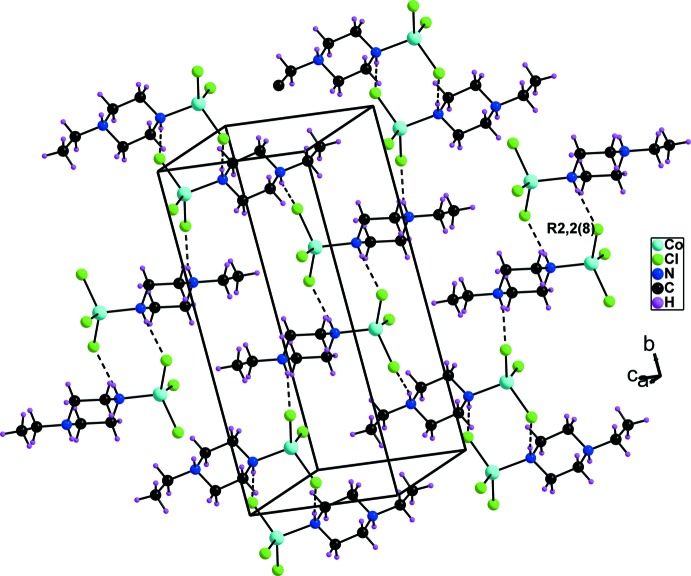
Perspective view of the crystal packing of the title complex. Hydrogen bonds are shown as dashed lines.

## supplementary crystallographic information

### 1. Comment

Piperazine (H_2_ppz) and its derivatives, are cyclic diamines possessing a non-planar six-membered ring analogous to cyclohexane with two basic nitrogen atoms in the 1,4 positions. As these nitrogen atoms are basic, piperazine (H_2_ppz) and its derivatives may coordinate metal ions as monodentate, bidentate or bidentate–chelate ligands. Several 1-methylpiperazine and 1,4-di- methylpiperazine platinum(II) complexes have been synthesized and characterized through X-ray diffraction (Ciccarese *et al.*, 1998). The cobalt complexes [CoCl_3_(HMe2ppz)] and [CoCl_3_(H_2_Meppz)] have shown interesting cytotoxic activity on human colon and carcinoma cells (Marzotto *et al.*, 2000). In order to explore possible biological applications and to gather further chemical and structural information on metal complexes capable of interacting selectively with nitrogen donors of DNA nucleobases, we have extended to cobalt(II) the study on the behaviour and coordinating properties of piperazine derivatives. This study includes the synthesis and structural characterization of the new complex, (C_6_H_15_N_2_)CoCl_3_ (I).

Complex I is present as a neutral zwitterionic (amphiionic) species. The negative charge of the –CoCl_3_ group is balanced by the positive charge at the ethylated N2 nitrogen atom of the 1-ethylpiperazin-1-ium ring. Zwitterionic complexes having the formula *MX*_3_LH (where *M* = Mn(II), Fe(II), Co(II), Ni(II), Cu(II), Zn(II); *X* = Cl, Br, I; LH = monoprotonated diamine), although rare, are already known. The six-membered piperazine ring in complex I possesses the characteristic chair conformation. The average of the Co(II)–Cl bond lengths, 2.261 Å, falls in the expected range. It is noteworthy that this Co(II)–Cl distance increases on increasing the number of chloride ions bonded to cobalt(II) atom, but also increases when the chloride ions are involved in strong hydrogen bonding. In the structure of I, the Co–Cl1, 2.2720 (8) Å, and Co–Cl3, 2.2691 (8) Å, distances are significantly longer than the Co–Cl2 distance, 2.2419 (10) Å , as the former are involved in strong hydrogen bonding while the latter is not. The Co(II)–N1 distance, 2.0686 (15) Å, is typical (Clemente *et al.*, 1999).

The structure consists of a neutral Co(II) complex with a 4-Etppz ligand bonded through the secondary nitrogen atom and three chloride ligands. The tertiary nitrogen atom is protonated balancing the overall charge (Fig. 1). Each complex undergoes two hydrogen bonding interactions as donors (N1—H1 and N2—H2) and two interactions as accpetors (Cl1 and Cl3). Hydrogen bonding occurs in two-dimensional sheets (Fig. 2). Dimer donor-acceptor hydrogen-bonding interactions (related by inversion) dominate the hydrogen bonding motif (*R*^2^_2_(8)) with additional single donor-acceptor hydrogen bonding interaction joining the dimer interactions. The same hydrogen-bonding pattern is found in the structure of the related [CoCl_3_(H_2_Meppz)] complex (Clemente *et al.*, 1999).

### 2. Experimental

The complex was synthesized by adding dropwise under stirring a blue-violet solution, previously prepared at 40°C, of anhydrous CoCl_2_ in 10 ml EtOH, to a 1-ethylpiperazine (HEtppz) solution dissolved in 5 ml EtOH in a molar ratio 1:1. After mixing, an aqueous hydrochloric acid solution was added to the blue powder compound and was stirred for 2 h. After filtration, the filtrate was allowed to stand at room temperature. Blue crystals were obtained by slow evaporation.

### 3. Refinement

Many hydrogen atoms were treated in calculated positions and refined in the model as riding with distances of C—H = 0.98 and 0.99 Å for the methyl and methylene groups, respectively, and with *U*_iso_(H) = k×*U*_eq_(C), k = 1.2. Hydrogen atoms H1 and H2 were located in the electron density map, and their positions were refined with *U*_iso_(H) = k×*U*_eq_(C), k =1.2.

### Crystal data

**Table:**

[Co(C_6_H_15_N_2_)Cl_3_]	*F*(000) = 572.00
*M**_r_* = 280.49	*D*_x_ = 1.591 Mg m^−^^3^
Monoclinic, *P*2_1_/*n*	Mo *K*α radiation, λ = 0.71075 Å
Hall symbol: -P 2yn	Cell parameters from 7042 reflections
*a* = 7.421 (3) Å	θ = 3.3–26.5°
*b* = 18.160 (7) Å	µ = 2.10 mm^−^^1^
*c* = 8.691 (4) Å	*T* = 173 K
β = 90.524 (7)°	Prism, blue
*V* = 1171.1 (8) Å^3^	0.32 × 0.13 × 0.13 mm
*Z* = 4	

### Data collection

**Table:**

Rigaku XtaLAB mini diffractometer	2099 reflections with *F*^2^ > 2σ(*F*^2^)
Detector resolution: 6.849 pixels mm^-1^	*R*_int_ = 0.025
ω scans	θ_max_ = 26.4°
Absorption correction: multi-scan (*REQAB*; Rigaku, 1998)	*h* = −9→9
*T*_min_ = 0.529, *T*_max_ = 0.761	*k* = −22→22
11015 measured reflections	*l* = −10→10
2399 independent reflections	

### Refinement

**Table:**

Refinement on *F*^2^	Secondary atom site location: difference Fourier map
*R*[*F*^2^ > 2σ(*F*^2^)] = 0.022	Hydrogen site location: inferred from neighbouring sites
*wR*(*F*^2^) = 0.050	H atoms treated by a mixture of independent and constrained refinement
*S* = 1.08	*w* = 1/[σ^2^(*F*_o_^2^) + (0.0195*P*)^2^ + 0.4*P*] where *P* = (*F*_o_^2^ + 2*F*_c_^2^)/3
2399 reflections	(Δ/σ)_max_ = 0.001
118 parameters	Δρ_max_ = 0.27 e Å^−^^3^
0 restraints	Δρ_min_ = −0.27 e Å^−^^3^
Primary atom site location: structure-invariant direct methods	

### Special details

**Table:**

Geometry. ENTER SPECIAL DETAILS OF THE MOLECULAR GEOMETRY
Refinement. Refinement was performed using all reflections. The weighted *R*-factor (*wR*) and goodness of fit (*S*) are based on *F*^2^. *R*-factor (gt) are based on *F*. The threshold expression of *F*^2^ > 2.0 σ(*F*^2^) is used only for calculating *R*-factor (gt).

### Fractional atomic coordinates and isotropic or equivalent isotropic displacement parameters (Å^2^)

**Table:**

	*x*	*y*	*z*	*U*_iso_*/*U*_eq_	
Co1	0.50887 (3)	0.124866 (12)	−0.13052 (2)	0.02446 (7)	
Cl1	0.62618 (6)	0.22726 (2)	−0.24155 (5)	0.03456 (11)	
Cl2	0.21414 (6)	0.13426 (3)	−0.07964 (6)	0.04714 (13)	
Cl3	0.58634 (6)	0.02448 (2)	−0.27061 (5)	0.03491 (11)	
N1	0.62169 (18)	0.11055 (8)	0.08596 (15)	0.0237 (3)	
N2	0.87397 (18)	0.13728 (7)	0.34217 (15)	0.0235 (3)	
C1	0.6034 (3)	0.17490 (9)	0.19013 (19)	0.0301 (4)	
C2	0.6795 (3)	0.15991 (10)	0.34991 (19)	0.0306 (4)	
C3	0.8943 (3)	0.07188 (9)	0.23786 (19)	0.0306 (4)	
C4	0.8130 (3)	0.08778 (10)	0.07959 (19)	0.0318 (4)	
C5	0.9516 (3)	0.12430 (10)	0.50161 (19)	0.0324 (4)	
C6	1.1522 (3)	0.10910 (10)	0.5011 (3)	0.0393 (5)	
H1A	0.4743	0.1880	0.1985	0.0361*	
H1B	0.6670	0.2176	0.1450	0.0361*	
H2A	0.6688	0.2048	0.4137	0.0367*	
H2B	0.6089	0.1203	0.3993	0.0367*	
H2	0.932 (3)	0.1733 (10)	0.3034 (19)	0.022 (5)*	
H3A	0.8333	0.0288	0.2837	0.0367*	
H3B	1.0237	0.0598	0.2272	0.0367*	
H4A	0.8835	0.1273	0.0298	0.0381*	
H4B	0.8227	0.0430	0.0151	0.0381*	
H5A	0.9283	0.1682	0.5660	0.0389*	
H5B	0.8890	0.0820	0.5490	0.0389*	
H6A	1.2141	0.1487	0.4461	0.0472*	
H6B	1.1749	0.0620	0.4497	0.0472*	
H6C	1.1974	0.1068	0.6073	0.0472*	
H1	0.566 (3)	0.0763 (11)	0.128 (3)	0.032 (6)*	

### Atomic displacement parameters (Å^2^)

**Table:**

	*U*^11^	*U*^22^	*U*^33^	*U*^12^	*U*^13^	*U*^23^
Co1	0.02308 (12)	0.02612 (12)	0.02421 (12)	−0.00175 (8)	0.00169 (8)	−0.00177 (9)
Cl1	0.0399 (3)	0.0238 (2)	0.0403 (3)	0.00338 (17)	0.01486 (18)	0.00187 (17)
Cl2	0.0227 (3)	0.0724 (4)	0.0464 (3)	−0.0018 (2)	0.00471 (18)	−0.0063 (3)
Cl3	0.0445 (3)	0.0260 (3)	0.0344 (3)	−0.00757 (17)	0.01151 (18)	−0.00670 (17)
N1	0.0221 (7)	0.0234 (8)	0.0257 (7)	−0.0032 (6)	0.0028 (6)	−0.0002 (6)
N2	0.0259 (7)	0.0209 (7)	0.0238 (7)	−0.0035 (6)	0.0013 (6)	0.0001 (6)
C1	0.0299 (9)	0.0289 (9)	0.0314 (9)	0.0056 (7)	−0.0011 (7)	−0.0051 (7)
C2	0.0278 (9)	0.0347 (10)	0.0294 (9)	0.0044 (7)	0.0039 (7)	−0.0085 (8)
C3	0.0312 (9)	0.0294 (9)	0.0312 (9)	0.0065 (7)	−0.0023 (7)	−0.0069 (7)
C4	0.0263 (9)	0.0406 (11)	0.0285 (9)	0.0069 (8)	0.0012 (7)	−0.0075 (8)
C5	0.0405 (10)	0.0347 (10)	0.0220 (9)	−0.0017 (8)	−0.0027 (7)	−0.0003 (7)
C6	0.0424 (11)	0.0400 (11)	0.0354 (10)	0.0069 (8)	−0.0107 (8)	0.0022 (8)

### Geometric parameters (Å, º)

**Table:**

Co1—Cl1	2.2720 (8)	C1—H1A	0.990
Co1—Cl2	2.2419 (10)	C1—H1B	0.990
Co1—Cl3	2.2691 (8)	C2—H2A	0.990
Co1—N1	2.0686 (15)	C2—H2B	0.990
N1—C1	1.485 (3)	C3—H3A	0.990
N1—C4	1.480 (3)	C3—H3B	0.990
N2—C2	1.502 (3)	C4—H4A	0.990
N2—C3	1.503 (3)	C4—H4B	0.990
N2—C5	1.514 (3)	C5—H5A	0.990
C1—C2	1.519 (3)	C5—H5B	0.990
C3—C4	1.525 (3)	C6—H6A	0.980
C5—C6	1.514 (3)	C6—H6B	0.980
N1—H1	0.83 (2)	C6—H6C	0.980
N2—H2	0.853 (18)		
			
Cl1—Co1—Cl2	113.57 (3)	H1A—C1—H1B	107.846
Cl1—Co1—Cl3	109.25 (4)	N2—C2—H2A	109.442
Cl1—Co1—N1	109.61 (5)	N2—C2—H2B	109.452
Cl2—Co1—Cl3	114.80 (2)	C1—C2—H2A	109.445
Cl2—Co1—N1	102.57 (5)	C1—C2—H2B	109.448
Cl3—Co1—N1	106.53 (5)	H2A—C2—H2B	108.051
Co1—N1—C1	114.64 (11)	N2—C3—H3A	109.507
Co1—N1—C4	112.42 (10)	N2—C3—H3B	109.508
C1—N1—C4	109.62 (13)	C4—C3—H3A	109.504
C2—N2—C3	110.19 (13)	C4—C3—H3B	109.503
C2—N2—C5	111.05 (13)	H3A—C3—H3B	108.076
C3—N2—C5	112.93 (13)	N1—C4—H4A	108.972
N1—C1—C2	112.39 (14)	N1—C4—H4B	108.974
N2—C2—C1	110.95 (14)	C3—C4—H4A	108.977
N2—C3—C4	110.70 (14)	C3—C4—H4B	108.982
N1—C4—C3	113.02 (14)	H4A—C4—H4B	107.779
N2—C5—C6	113.06 (14)	N2—C5—H5A	108.969
Co1—N1—H1	107.3 (13)	N2—C5—H5B	108.974
C1—N1—H1	105.7 (14)	C6—C5—H5A	108.963
C4—N1—H1	106.6 (14)	C6—C5—H5B	108.971
C2—N2—H2	107.2 (12)	H5A—C5—H5B	107.768
C3—N2—H2	108.2 (12)	C5—C6—H6A	109.474
C5—N2—H2	107.0 (11)	C5—C6—H6B	109.466
N1—C1—H1A	109.127	C5—C6—H6C	109.477
N1—C1—H1B	109.120	H6A—C6—H6B	109.472
C2—C1—H1A	109.128	H6A—C6—H6C	109.470
C2—C1—H1B	109.129	H6B—C6—H6C	109.468
			
Cl1—Co1—N1—C1	55.30 (9)	C4—N1—C1—C2	−55.55 (16)
Cl1—Co1—N1—C4	−70.78 (9)	C2—N2—C3—C4	54.82 (16)
Cl2—Co1—N1—C1	−65.66 (8)	C3—N2—C2—C1	−55.64 (16)
Cl2—Co1—N1—C4	168.27 (7)	C2—N2—C5—C6	−174.02 (12)
Cl3—Co1—N1—C1	173.39 (7)	C5—N2—C2—C1	178.47 (12)
Cl3—Co1—N1—C4	47.31 (9)	C3—N2—C5—C6	61.63 (17)
Co1—N1—C1—C2	176.93 (8)	C5—N2—C3—C4	179.64 (12)
Co1—N1—C4—C3	−176.03 (9)	N1—C1—C2—N2	56.87 (17)
C1—N1—C4—C3	55.22 (17)	N2—C3—C4—N1	−55.84 (18)

### Hydrogen-bond geometry (Å, º)

**Table:**

*D*—H···*A*	*D*—H	H···*A*	*D*···*A*	*D*—H···*A*
N2—H2···Cl1^i^	0.854 (19)	2.347 (18)	3.1794 (16)	165.4 (15)
N1—H1···Cl3^ii^	0.83 (2)	2.49 (2)	3.3192 (17)	176.0 (17)

Symmetry codes: (i) *x*+1/2, −*y*+1/2, *z*+1/2; (ii) −*x*+1, −*y*, −*z*.
